# The Bacterial Microflora of Fish

**DOI:** 10.1100/tsw.2002.137

**Published:** 2002-03-05

**Authors:** B. Austin

**Affiliations:** Department of Biological Sciences, Heriot-Watt University, Riccarton, Edinburgh EH14 4AS, Scotland, UK

**Keywords:** bacteria, fish, microflora, methods, digestive tract, gills, skin, population size, taxonomy, biodiversity, luminescence, degradative ability, effect of antibiotics, polymers, enzymes, spoilage

## Abstract

The results of numerous studies indicate that fish possess bacterial populations on or in their skin, gills, digestive tract, and light-emitting organs. In addition, the internal organs (kidney, liver, and spleen) of healthy fish may contain bacteria, but there is debate on whether or not muscle is actually sterile. The numbers and taxonomic composition of the bacterial populations often reflect those of the surrounding water. The role of the bacteria includes the ability to degrade complex molecules (therefore exercising a potential benefit in nutrition), to produce vitamins and polymers, and to be responsible for the emission of light by the light-emitting organs of deep-sea fish. Taxa, including *Pseudomonas*, may contribute to spoilage by the production of histamines in fish tissue.

## INTRODUCTION

It is a common fault that research publications purporting to consider the microflora of fish focus only on the bacteria (typically the aerobic heterotrophic bacterial component[1]) to the exclusion of eukaryotes. Anaerobic bacteria have been comparatively neglected[2,3,4,5], possibly reflecting the need for more exacting techniques, although there is increasing evidence that such organisms occur in large numbers especially within the digestive tract of freshwater and marine fish[2].

This article will synthesise the available information on fish-associated bacteria, focusing on the numbers, nature, and role of bacteria on or in healthy finfish. Aspects of fish pathology will be ignored, as a wealth of information sufficient to fill several textbooks already exists[6]. However, it is apparent that some pathogens may be found on healthy fish in the absence of disease. It is questionable whether such associations represent the asymptomatic carrier state of the disease cycle, a preliminary colonisation step prior to pathogenesis, or commensalism-synergism. For example, *Flavobacterium psychrophilum,* the causal agent of coldwater disease (of salmon) and rainbow trout fry syndrome, has been found in the kidney, spleen, brain, ovarian fluid, unfertilised eggs, and milt of healthy Baltic salmon (*Salmo salar*)[7].

It is apparent that fish are continuously exposed to the microorganisms present in water and in sediment. These organisms will undoubtedly influence the microflora on external surfaces, including the gills, of fish. Similarly, the digestive tract will receive water and food that is populated with microorganisms. Certainly, colonisation may well start at the egg and/or larval stage, and continue with the development of the fish[8]. Thus, the numbers and range of microorganisms present in the eggs, on food, and in the water, will influence the microflora of the developing fish. Also, it is recognised that, to some extent, it is possible to manipulate the microflora of the developing fish by use of probiotics, i.e., live microbial food supplements, which may colonise the digestive tract for short or prolonged periods[9].

From the published literature, it may be deduced that there are three likely scenarios for the fate of bacteria coming into contact with fish:

1. The organisms from the environment around the fish may become closely associated with and even colonise the external surfaces of the fish. There may be accumulation of the organisms at sites of damage, such as missing scales or abrasions[10].
2. The organisms may enter the mouth with water[8] or food and pass through and/or colonise the digestive tract[10].
3. The organisms coming into contact with fish surfaces may be inhibited by the resident microflora or by natural inhibitory compounds present on the fish[10].

The overriding problem concerns whether or not it is possible to differentiate members of the indigenous (fish) microflora from transients, which could be in the water film around fish or in water/food within the digestive tract. Unfortunately, it is apparent that most publications do not address this issue. Yet this is not so unusual insofar as similar arguments have centred on the nature of the true microflora of other habitats, e.g., the distinction between microbial populations of the rhizoplane (root surface) vs. the rhizosphere (habitat around roots), and of the phylloplane (leaf surface) vs. the phyllosphere (habitat around leaves).

It is recognised that extraneous bacteria are capable of surviving in fish. For example, the faecal indicator organism, *Escherichia coli,* has been found to survive and even multiply in the digestive tract of rainbow trout (*Oncorhynchus mykiss*) after ingestion via contaminated food[11].

## RESEARCH ACTIVITIES

Research has focused on six principal aspects of the microbiology of fish:

- The microbiology of the surface (including gills)
- The populations in the digestive tract
- The possible presence of bacteria in muscle and the internal organs of healthy fish
- The microflora of eggs
- The presence and role of bacteria associated with the light-emitting organs, particularly of deep-sea fish
- The bacterial populations in food

As a simplification, publications have tended to emphasise either quantitative or qualitative aspects or the supposed role of the organisms on/in fish. It is unusual for research articles to address more than one of these aspects.

## METHODS USED TO STUDY FISH-ASSOCIATED BACTERIA

### Dilution and Spread-Plating Techniques

There has been a tendency for scientists to study fish-associated bacteria using only traditional, comparatively simple techniques, such as dilution or spread plating, incorporating media and incubation conditions often of dubious relevance. As an example, to isolate bacteria from the skin, the surface of the fish may be swabbed over an indeterminate area, and the inoculum spread over the surface of nutrient-rich medium, such as tryptone soya agar (TSA[12]), with incubation at 15–25$^{o}$C for 7–14 days. A disadvantage of using swabbing techniques is that it is difficult to equate the data with a defined unit of measurement. In addition, the relevance of the resulting data for ascertaining the indigenous bacterial populations on the surface of fish is questionable. Yet such approaches have been common until fairly recently.

Criticism may also be levied at the time taken between sample collection and examination, which may often be measured in hours or days. It is not uncommon for whole or parts (e.g., the digestive tract) of fish to be transported on ice, cooled or at ambient temperature, to distant laboratories for examination. Sections or the entire digestive tract, together with the contents, have then been homogenised, in which case it is impossible to decide from the data if the resulting bacteria have originating from the food particles, lumen, and/or the wall. Some workers have studied the bacterial populations of the digestive tract by swabbing the anus and faeces[10]. Unfortunately, most scientists do not consider whether or not there may be significant changes in the microflora during the period from collection of the fish to microbiological examination.

As a final criticism, it is noted that the proportion of the bacterial microflora contributing to the colony count is rarely considered in quantitative-type studies. Circumstantial evidence suggests that populations deduced from colony counts on agar plates greatly underestimate the total microflora likely to present. Nevertheless, a recent study considered that at much as 50% of the microflora from the intestine of rainbow trout produced colonies on TSA[12] — thus, a 50% error might be inferred!

Some comparisons between methods have been conducted. For example, gentle rinsing techniques have been evaluated and compared to excision and homogenisation with a stomacher to recover bacteria from the surface of fish[13]. In this case, stomaching was regarded as superior for the enumeration of total bacterial populations, but rinsing was better for rainbow trout than striped bass (*Morone saxatilis*)[13].

### Microscopic Techniques

Microscopic techniques have found increasing use in the study of fish microflora, and include direct microscopic counts by light[12,14] and electron microscopy[14,15,16]. These have been used to visualise the organisms present, particularly in the digestive tract.

### Automated Direct Epifluorescent Filter Technique

An automated direct epifluorescent filter technique instrument, COBRA, has been evaluated, and offers promise for enumerating bacterial populations[17].

### Molecular Techniques

Molecular methods are increasingly being used. For example, numerous publications have discussed the sequencing of16S rRNA genes[12,18,19,20]. Also, microplate hybridisation has been successful at identifying aeromonads in the digestive tract of freshwater fish[21].

## QUANTITATIVE ASPECTS OF THE BACTERIAL MICROFLORA

### Surface Populations

Most workers have opted for the comparatively easy approach of studying the aerobic heterotrophic bacteria populations, with data suggesting that the numbers of bacteria on the surface of fish approximate those of the surrounding water. Yet, in retrospect, it is apparent that the units of measurement between water (populations $ml^{-1}$) and fish surfaces (populations $cm^{-2}$) are very different, and comparisons are not especially helpful.

Scrutiny of the available literature suggests that fish have only low bacterial populations on the skin. For example, Atlantic salmon (*Salmo salar*) from the U.K. were reported to possess populations of $10^2$–$10^3$ culturable bacteria $cm^{-2}$ of skin[22], whereas rainbow trout from Turkey contained bacterial populations of $10^1$–$10^7$ $g^{-1}$[23]. However, it should be emphasised that the relevance of using weight as a unit of measurement to estimate bacterial populations on skin is debatable. Interestingly, freshly caught mullet (*Mugil cephalus*), whiting (*Sillago ciliato*), and flathead (*Platycephalus fiscus*) from Australia were reported to have seemingly higher populations of $4 \times 10^3$ to $8 \times 10^4$ bacteria $cm^{-2}$[24]. Not surprisingly, there are data suggesting that the bacterial population size reflects the level of water pollution, i.e., higher counts are present on fish in polluted waters[25]. Also, there is some evidence that the population of aerobes exceeds that of the anaerobes[26].

Bacterial populations of skin
$10^2$ to ~$10^4$ $cm^{-2}$

Overall, these low counts, which to some extent have been supported by scanning electron microscopic evidence[10], indicate that only a minute area of the fish surface is populated with bacteria. However, it is conceded that the preparation for scanning electron microscopy may well have removed some organisms from the skin. Thus, it could be inferred that the surface microflora is only loosely associated with fish skin. Coincidentally, this low level of "colonisation" on fish skin corresponds well to that of other habitats, such as the leaf surface of terrestrial plants[27].

### Gills

Gill tissue has been found to harbour high bacterial populations, e.g., up to $10^6$ bacteria $g^{-1}$ of gill tissue[28].

### Eyes

There is anecdotal evidence that the eyes of healthy fish are devoid of bacterial colonisation[10].

### Muscle and the Internal Organs

Muscle has been considered by some to be sterile[29], whereas other investigators have reported the presence of bacteria[30]. Also, some workers have found bacteria in the kidney and liver of healthy fish, i.e., turbot (*Scophthalmus maximus*)[31].

### Digestive Tract

A consensus view is that dense bacterial populations occur in the digestive tract (i.e., populations of up to ~$10^8$ heterotrophs $g^{-1}$ [32,33,34,35]) and ~$10^5$ anaerobes $g^{-1}$[3,32,33] have been reported with numbers appearing to be much higher than those of the surrounding water. For example, by including contents with the intestine, maximal bacterial populations of $2 \times 10^7$ colony-forming units $g^{-1}$ were recorded in the pinfish (*Lagodon rhomboides*)[36]. Moreover, counts of $1.1 \times 10^6$ to $3.6 \times 10^8$ bacteria were recorded for the intestinal contents of deep-sea fish[35], when it was noted that more cultures were obtained at *in situ* (barophilic), rather than atmospheric, pressure[35]. Also, some differences have been considered to reflect seasonality, i.e., with maximum and minimum counts occurring in summer and winter, respectively[37]. Indeed, an effect of water temperature on the size of the microflora of pike perch (*Stizostedion lucioperca*) has been reported[38]. In another study, seasonal variation was attributed to the monsoon season, with maximal and minimal populations in green chromides (*Etroplus suratensis*) and orange chromides (*E. maculates*) corresponding with postmonsoon (September to December) and premonsoon (January to April), respectively[39].

Differences in population size have been detected in specific regions of the digestive tract. Thus, an estimation of aerobic heterotrophs in the digestive tract of yellowtail (*Seriola* sp.) revealed counts of $2 \times 10^4$ bacteria $g^{-1}$, $2.5 \times 10^5$ bacteria $g^{-1}$, and $6.5 \times 10^4$ to $5.9 \times 10^6$ bacteria $g^{-1}$ in the pyloric caeca, stomach, and intestine, respectively[40]. Parallel data were published separately[38], when the presence of $5.5 \times 10^3$ to $5.0 \times 10^4$ colony-forming units (cfu) of aerobic heterotrophic bacteria $g^{-1}$ and $1.0 \times 10^4$ to $1.0 \times 10^7$ cfu aerobic heterotrophic bacteria $g^{-1}$ were found in the stomach and intestine of pike perch, respectively. However, higher populations were noted in the digestive tract of juvenile compared with adult farmed Dover sole (*Solea solea*), with $5.2 \times 10^5$, $8.0 \times 10^5$, and $9.8 \times 10^6$ aerobic heterotrophs $g^{-1}$ recovered from the stomach-foregut, midgut, and hindgut-rectum (of juvenile fish), respectively[41]. It should be emphasised that anaerobes ($7.1 \times 10^5$ anaerobic bacteria $g^{-1}$) have been found in addition within the intestines[40].

Following a familiar theme, it was observed that there was an increase in bacterial populations, especially of adherent organisms, along the digestive tract of herring (*Clupea harengus*) larvae[14].

Some differences have been noted according to the feeding pattern of fish. Thus, detritivorous fish possessed higher bacterial populations than filter feeders[42]. Of course, it is likely that many organisms in the digestive tract will have been derived from the food. In this connection, it was found that there were between $10^3$ and $10^7$ aerobic heterotrophs $g^{-1}$ in commercial fish food in North America[43], whereas comparable data from Japan indicated counts of $1.8 \times 10^3$ to $8.0 \times 10^5$ bacteria $g^{-1}$[44].

Electron microscopy has substantiated the presence of high bacterial populations in the digestive tract. In particular, scanning and transmission electron microscopy demonstrated the presence of large populations of ovoid and rod-shaped bacterial-like objects in association with the microvillous brush borders of enterocytes of Arctic char (*Salvelinus alpinus*)[16]. Also, bacteria were observed at and between the tips of microvilli, and rod-shaped bacteria were seen between the microvilli of common wolffish (*Anarhichas lupus*)[15]. Evidence has pointed to endocytosis of bacteria by epithelial cells in the pyloric caeca and midgut[16].

Bacterial populations in the digestive tract
$\sim 10^8$ aerobic heterotrophs $g^{-1}$
$\sim 10^5$ anaerobic $g^{-1}$

## Fish Eggs and Larvae

Fish eggs may be populated by high numbers of bacteria, with $10^3$–$10^6$ bacteria $g^{-1}$ reported for salmonid eggs[45]. There is evidence that adhesion and colonisation of the egg by bacteria occurs within a few hours of fertilisation[46]. Undoubtedly, these organisms — and those of the food and surrounding water — are important for the establishment of a microflora in the digestive tract of fish larvae. Incidentally, the digestive tract of newly hatched larvae contains scant bacterial populations, but is quickly colonised[47].

## TAXONOMY (BIODIVERSITY)

Approaches have gone from the traditional[48], through numerical taxonomy studies involving large numbers of isolates (e.g., 197 cultures investigated in one study[49]; 473 isolates studied in another[1]), to molecular approaches (e.g., partial sequencing of the 16S rRNA gene[12]). Sometimes, the phenetic approach has centered on the use of rapid systems, such as BIOLOG or MIDI[26,50]. It is encouraging that some comparative studies have pointed to congruence between phenotypic and molecular analyses[12]. Overall, it would appear that narrow-based studies focusing on a limited number of bacterial groups are often more successful than those that attempt to be broad-based, trying to consider all of the bacteria from fish. From the published literature, it is apparent that there are many similarities between the bacterial populations in fish and water[23,29,30,48,51,52,53,54,55,56,57] .

### Surface Microflora

The bacteria from the surface of freshwater fish have been reported to include *Acinetobacter johnsonii*[58], aeromonads (notably *Aeromonas hydrophila, A. bestiarum*, *A. caviae*, *A. jandaei*, *A. schubertii,* and *A. veronii* biovar sobria[59])*, Alcaligenes piechaudii, Enterobacter aerogenes, Escherichia coli, Flavobacterium*[25], *Flexibacter* spp., *Micrococcus luteus, Moraxella* spp., *Pseudomonas fluorescens,* psychrobacters[58], and *Vibrio fluvialis*[23,56,60]. To some extent, the presence of aeromonads reflected whether or not the water in which the fish occurred was polluted or clean[59].

Bacteria, typical of those in seawater, have been recovered from the surface of marine fish and include *Acinetobacter calcoaceticus, Alcaligenes faecalis, Bacillus cereus, B. firmus, Caulobacter,* coryneforms, *Cytophaga/Flexibacter, E. coli, Hyphomicrobium vulgare, Lucibacterium (Vibrio) harveyi, Photobacterium angustum, P. logei, Prosthecomicrobium, Pseudomonas fluorescens, P. marina*, and *Vibrio* spp. (including *V. albensis, V. anguillarum, V. splendidu*s biotype I, *V. fischeri, V. ordalii*, and the new species *V. scophthalmi* on the surface of turbot)[1,54,55].

As a result of a detailed numerical taxonomic study of Gram-negative oxidase-positive bacteria recovered from sharks, the dominance of vibrios was noted, with representatives including *V. harveyi (V. carchariae)*, and *V. alginolyticus*. Other groups included *Aeromonas, Photobacterium* (including *P. damselae* and *P. damselae* subsp. *piscicida*), *Alteromonas, Plesiomonas shigelloides, Moraxella,* and *Neisseria*[49].

### Gill Microflora

Yellow-pigmented Gram-negative rods, especially *Cytophaga* spp., dominate on gills[28]. Aeromonads, coryneforms, enterobacteria, Gram-positive cocci, pseudomonads, and vibrios have also been recovered from the gills of healthy juvenile rainbow trout[57].

Gills of marine fish accommodate *Achromobacter, Alcaligenes, Bacillus, Flavobacterium,* and *Micrococcus*[61] and yellow-pigmented bacteria, loosely associated with *Chryseobacterium-Flavobacterium-Flexibacter-Cytophaga*[62].

## Microflora in the Digestive Tract

Initially in the sac fry, only a few taxa (coryneforms and *Pseudomonas*) occur within the digestive tract[45]. It is likely that some bacteria become ingested at the yolk-sac stage, leading to the establishment of an initial intestinal microflora[46]. In addition, it has been reported that bacterial colonisation of the digestive tract of turbot larvae coincided with the start of feeding, when the microflora was dominated by *Aeromonas* and *Vibrio*[63]. In an investigation of the intestinal microflora of larval sea bream (*Dicentrarchus labrax*) and sea bass (*Sparus aurata*), it was observed that when the larvae were fed with rotifers, there was a high incidence of *V. anguillarum*, *V. tubiashii*, and nonvibrio groups[64]. However, feeding with *Artemia* led to the recovery of mostly *V. alginolyticus, V. proteolyticus, V. harveyi,* and *V. natriegens*[64]. It was concluded from these experiments that the fluctuations in the dominant components of the microflora reflected the bacteria in the live feed. Indeed, the dominance of vibrios was not recorded until the end of the larval stage[64]. The comparative lack of diversity in larvae continues into older fish, and it has been suggested that the flora may be subjected to as-yet-undescribed selective effects leading to a restricted number of taxa being present[48,65,66,67,68].

A comparatively wide range of taxa have been associated with the digestive tract of adult freshwater fish, and include *Acinetobacter, Enterobacter, Escherichia, Klebsiella, Proteus, Serratia*[32], *Aeromonas*[32,33,37,57,69,70] — isolates have been identified by microplate hybridization as *A. caviae, A. hydrophila, A. jandaei, A. sobria*, and *A. veronii*[21] — *Alcaligenes*, *Eikenella*[4], *Bacteroides* [3,71,72], *Citrobacter freundii*[29], *Hafnia alvei*[69], *Cytophaga/Flexibacter*[57], *Bacillus, Listeria, Propionibacterium, Staphylococcus*[29], *Moraxella*[38], and *Pseudomonas*[4,29,57,68]. In one study involving pike perch, it was concluded that *Moraxella* and *Staphylococcus* were unique to the habitat when compared with the digestive tract of other fish species[38].

Modern phenetic and molecular-based studies have indicated variability in the intestinal microflora of rainbow trout reflecting the fish farm of origin[12], with analyses revealing the dominance of the gamma subclass (*Citrobacter, Aeromonas,* and *Pseudomonas*) and beta subclass of Proteobacteria, and Gram-positive bacteria with a low G + C-content of the DNA (*Carnobacterium*)[12].

The digestive tract of adult marine fish has been reported to contain *Aeromonas*[69], *Alcaligenes*[41,73], *Alteromonas*[14], *Carnobacterium*[74], *Flavobacterium*[41,73], *Micrococcus*[41], *Photobacterium*[41,73], *Pseudomonas*[48,73], *Staphylococcus*[41], and *Vibrio*[14,41,48,51,53,68,70,73], including *V. iliopiscarius*[75].

Special groups, such as large (gigantobacteria) symbiotic bacteria, have been observed in the digestive tract of surgeonfish from the Red Sea and Indo-Pacific Region[76]. Also, using a methanogen-specific nested polymerase chain reaction, methanogens have been detected in the digestive tract and faeces of flounder (*Platichthys flesus*) from the North Sea[20]. Indeed, in this study, 16S rDNA sequences revealed 97.6–99.5% similarity to the archaea representative *Methanococcoides methylutens*[20].

Carnobacteria are common on/in fish, particularly in the digestive tract[77]. To date, studies have emphasised the taxonomy of the organisms[77], highlighting the presence of *C. piscicola* [77,78] and *C. piscicola*–like bacteria[79], and their role as putative probiotics for use in aquaculture. Other lactic-acid bacteria present in the epithelial mucosa have been equated with *Lactobacillus plantarum*, *Leuconostoc mesenteroides,* and *Streptococcus* spp.[77]. In a separate investigation, *Lactobacillus, Enterococcus durans, Lactococcus, Vagococcus*, *C. divergens,* and *C. piscicola* were recovered from freshwater fish, notably brown trout (*Salmo trutta*) and characterised phenotypically with numerical analyses[80]. A new species, *C. inhibens,* was recovered from the intestine of Atlantic salmon, and demonstrated antibacterial activity against fish pathogens, notably *Aeromonas salmonicida* and *Vibrio anguillarum*[81].

### Diets

Aeromonads, *Bacillus,* pseudomonads, and *Staphylococcus* dominate in diets[25,44].

### Eggs

Healthy eggs are populated by *Cytophaga/Flavobacterium* and, to a lesser extent, *Pseudomonas*[45,82], reflecting the organisms present in water[46].

### Internal Organs

The liver and kidney of healthy turbot have been found to be populated by mostly *Pseudomonas* and *Vibrio,* including *V. fischeri, V. harveyi, V. pelagius,* and *V. splendidus*[31]. Similarly, *Shewanella* spp. have been recovered from the internal organs[83]. The reasons for the presence of some of these bacteria are unclear. Moreover, it is speculative whether or not the fish are at the earliest stage of an infection cycle.

### Human Pathogens Recovered from Fish Tissue

Attention has focused on the presence of potential human pathogens in and around fish. For example, *Plesiomonas shigelloides* has been cultured from the digestive tract of pike[5]. Similarly, *Staphylococcus aureus* and *V. mimicus* have been isolated repeatedly from striped bass reared in flow-through and recirculating systems[84]. *V. cholerae* was recovered from presumably healthy sharks[49]. *V. vulnificus,* enumerated by the most probable number technique with serological identification, has been found in the contents of the digestive tracts of numerous fish from the U.S. Gulf Coast[85]. In this study, a seasonal fluctuation was recorded with minimum and maximum numbers occurring in winter and April to October, respectively. Indeed, the highest populations of *V. vulnificus* ($10^8$ bacterial 100 $g^{-1}$) were associated with the gut contents of bottom-feeding fish, especially those that consumed molluscs and crustacea[85]. In contrast, the plankton-feeding fish contained $10^5$ cells of *V. vulnificus* 100 $g^{-1}$. Overall, it was apparent from this study that the incidence of *V. vulnificus* was comparatively uncommon in offshore fish, instead being restricted to those specimens from estuaries, i.e., closer to shore[85]. In contrast, there has not been any evidence of *Listeria monocytogenes*[5]*, Salmonella,* or *Yersinia enterocolitica*[26,50].

## THE ROLE OF FISH BACTERIA

Although the relative numbers and types of bacteria associated with healthy fish are interesting, it is the role of these bacteria that is of importance. However, the information is generally patchy. For a start, it is relevant to inquire whether fish-associated bacteria are active metabolically or could some be inactive-dormant-nonculturable[86]. By piecing together various data, it becomes apparent that components of the bacterial microflora of fish have been associated with numerous functions, including: (1) the production of friction-preventing polymers (bacteria on fish skin, perhaps, important for the movement of fish through the water column[87]), (2) eicosapentaenoic acid (intestinal bacteria[88]), (3) the degradation of complex molecules, including starch (amylase production by intestinal bacteria[89,90]), cellulose[36], phospholipids (intestinal bacteria[91]), chitin, and collagen[41,90], and (4) the production of neuraminidase (in *Photobacterium damselae,* from the intestines of coastal fish[92]) and vitamins (e.g., vitamin $B_{12}$, which may be of value to the host[71,93,94,95]. Some taxa, such as *Pseudomonas,* have been implicated as causes of fish spoilage[96,97] by the production of histamines[98,99], principally during storage of fish[61].

Thus, it is likely that bacteria are often beneficial by contributing to the nutritional processes of fish, namely by degrading complex molecules in the digestive tract[41] and by producing vitamins[71].

## Luminescent Bacteria

Luminous bacteria, principally *Photobacterium*[100,101], including *P. phosphoreum* and *P. leiognathi*[19], organisms related to *P. phosphoreum* as determined by 16S rRNA sequencing[18], and *Vibrio* spp.[101], including *V. fischeri*[86,102,103], are responsible for the light-emitting properties of fish from ten families and five orders[19,104,105]. In addition, obligately symbiotic luminous bacteria that have been equated by 16S rRNA analyses as new species of *Vibrio* have been found in members of the beryciform family Anomalopidae and nine families in the lophiiform suborder Ceratioidei[19]. Generally, luminous bacteria are extracellular, and appear to be tightly arranged in tubules with communication with the exterior of the light-emitting organ[101]. These tubules release bacteria into the digestive tract of the host and thus into the surrounding seawater, where the released organisms are viable and culturable, and may well contribute to the planktonic microbial populations[101]. Superficially, it would seem that this work has been largely substantiated by others, who have also recognised the presence of luminous bacteria, namely *Photobacterium* (*P. phosphoreum*) and *V. harveyi,* in the digestive tracts of some marine fish[106]. However, it should be emphasised that many fish without light-emitting organs have also been found to possess luminescent bacteria in their intestines[106]. Therefore, light-emitting organs are clearly not always the source of luminous bacteria in the digestive tract or, for that matter, in seawater.

The production of light by the light-emitting organ is a direct function of synergism or symbiosis between luminous bacteria and fish. There is some evidence that luminous bacteria pass from the adults to offspring[107]. In particular, it was found that offspring from spotnape ponyfish (*Leiognathus nuchalis*) eggs, which were hatched in the absence of adults, did not develop luminescence activity[107]. Conversely, juvenile fish developed bioluminescence within 48 h of contact with adults or inoculation with a homogenate of the adult light-emitting organs[107]. From this work, the inference was that juvenile fish became infected with symbiotic luminous bacteria from the light-emitting organ of adult fish, thereby gaining the ability to become bioluminescent[107].

Luminous bacteria in the intestine appear to be involved in chitin degradation, and may therefore have a role in the digestion of complex molecules[108]. Also, some luminous bacteria have been attributed with the ability to produce histamine, and could, therefore, be involved in fish spoilage[109].

## Production of Inhibitory Compounds

Some bacteria produce inhibitory compounds, particularly in the digestive tract, and may be responsible for controlling the colonisation of potential pathogens in fish[79]. For example a *Vibrio* sp. recovered from the intestine of a spotnape ponyfish in Japanese coastal waters inhibited the causal agent of pasteurellosis/pseudotuberculosis, i.e., *P. damselae* subsp. *piscicida*[110]. Specifically, the inhibitory compound was heat-labile and proteinaceous, with a molecular mass of <5 kDa, and was considered to be possibly a bacteriocin or a bacteriocin-like substance[110].

Similarly, bacteria were isolated and found to be capable of inhibiting growth of pathogenic *Vibrio* sp. from the digestive tract of halibut (*Hippoglossus hippoglossus*) larvae[111]. Here, the fraction of pathogen inhibitors among the total number of isolates ranged between 0–100% (first feeding) and 0–66% (weaning). All antagonists were Gram-negative rods, most of which were fermentative, and produced catalase and oxidase, being equated with *Aeromonas* and *Vibrio*[111].

Using a double agar layer method, 940 aerobic and anaerobic isolates obtained from the digestive tract of river fish, water, and sediment in Japan were examined for antagonism[72]. Some of the isolates (i.e., *Bacteroides* type A and other Bacteroidaceae representatives) from the digestive tract inhibited the target organisms, which included *A. hydrophila, A. salmonicida, Escherichia coli,* and *Staphylococcus aureus.* The implication of the data was that these antagonistic bacteria may well influence the composition of the microflora in the digestive tract by the production of inhibitory compounds[72]. In another study by the same group, it was reported that, of 1,055 intestinal bacteria derived from 7 coastal fish in Japan, 28 isolates (2.7% of the total) inhibited the human and eel pathogen *V. vulnificus*[112]. Thus, marked inhibition was displayed by 15 isolates, comprising 11 Vibrionaceae representatives, 3 coryneforms, and 1 *Bacillus* strain NM 12; the latter demonstrated the most profound antimicrobial activity, and was therefore chosen for detailed study[112]. This revealed that one of the inhibitory compounds, which was determined to be a heat labile siderophore of <5 kDa molecular weight, inhibited the growth of 227 out of 363 (62.5% of the total) intestinal bacterial cultures derived from 7 fish[112]. Others have achieved this level of success. For example, of >400 bacteria recovered from turbot, 89 inhibited the growth of the fish pathogen *V. anguillarum*[113]. Similarly, of >400 isolates from the intestine and the external surface of farmed turbot, 28% (mostly from the digestive tract) inhibited *A. salmonicida, A. hydrophila,* and *V. anguillarum*[114].

### Effect of Antimicrobial Compounds on the Microflora

When fish become exposed to antimicrobial compounds, there will undoubtedly be an impact on the composition of the microflora and on antibiotic resistance patterns[14,115,116]. This, in turn, may impact upon the transmission of antibiotic resistance, such as via R-factors[116], to other bacteria, and perhaps of significance to humans.

## CONCLUSIONS

Fish possess a diverse array of bacterial taxa, often reflecting the composition of the microflora of the surrounding water. It is argued that the role of many of these fish-associated bacteria is unclear, and future work should be directed at this aspect.

## BIOSKETCH

**Brian Austin**, Sc., Ph.D., D.Sc., I.L.T.M, is Professor of Microbiology at Heriot-Watt University in Edinburgh, Scotland. His research interests are in taxonomy (including modern methods of data analysis), ecology (notably the fate of aeromonads and vibrios in the aquatic environment), diagnosis (principally the development of rapid diagnostic methods), pathogenesis (current emphasis is on atypical *Aeromonas salmonicida* and *Vibrio harveyi*) and control (by the development of vaccines and probiotics) of bacterial fish pathogens of relevance to aquaculture. Marine biotechnology is also an area of interest, with emphasis on the development of novel antibiotics and enzymes from (marine) bacteria. Prof. Austin is a Fellow of the American Academy of Microbiology; FSRA; and Guest Professor, Ocean University of Qingdao.
